# Some Outstanding Points in Clinical Medicine

**Published:** 1919-09-06

**Authors:** 


### WAR EXPERIENCE AND CIVIL PRACTICE.

### Some Outstanding Points in Clinical Medicine.

Although it is certain that much of our war experience will fortunately, perhaps, be of relatively little direct application in civil practice, yet in a number of medical diseases we have gained much invaluable knowledge. In an excellent address recently delivered by Sir William Hale-White, the author summarises the situation by taking the various systems of the body and concisely both tracing and illustrating the changes which have recently come about in our generally accepted ideas. We venture to draw attention to certain of his more essential paragraphs as probably providing the key to many of the paths which both medical training and practice must follow in the near future.

### The Heart.

A vexed question has been the determination of the heart's fitness for muscular work, particularly from recruiting and invaliding standpoints. Our knowledge of actual valvular disease may not have been increased, but it is certainly nowadays more widely diffused, and it is more frequently remembered that what really matters is not so much the noise we hear on auscultation, but whether the heart is doing its work efficiently. Often the louder a murmur is the better, for a loud murmur may mean that the cardiac muscle is strong. It is impossible here to quote the full directions provided for Medical Recruiting Boards, where it was laid down that if a man's life and occupation did not prove his capability for physical exertion, his reaction should be tested by certain trial exercises, and if he has not already met them, the reader can readily refer to the official publications. The point is that dilated heart has frequently been diagnosed on insufficient grounds, and a too serious import attached to pulse irregularity, due merely to extra systoles. The danger of overstating the weakness of a heart lies not only in the unnecessary restrictions placed upon the patient, but also in the fixed idea of heart disease and its attendant disadvantages and misery. As Sir W. Hale-White says, the heart must be judged by the work it can do; it is the duty of the doctor to determine this and advise accordingly.

### Epilepsy.

One is liable to forget that about one in every 1,500 persons in this country is an epileptic, and that the proportion is highest at the common recruiting age. The epileptic was particularly unfit to withstand the nervous stress of war. To himself and others there were the risks coincident with insanity, the violent motor fits, and the frequent automatism. Yet an enormous number of mistakes have been made concerning this condition, even more regrettable for the unnecessary expense they have brought on the country. *Petit mal* was extremely likely to be overlooked, but by no means free from danger. Severe fits frequently developed. The patient was liable to mental aberration, to weakmindedness, and is quite unfitted for a soldier. Loss of consciousness may be but momentary or even incomplete, and, as the author points out, the lesson for civil practice that our war experience teaches is that we should be more on the outlook for epilepsy and more careful in our occupational disposal of these cases.

### The Lungs and Pleura.

Although wounds in this region are unusual in civil practice, we may note that experience with hæmothorax cases has emphasised the necessity for early draining the pleural cavity of unsterile fluid, and even if it is sterile this drainage helps the lung to expand more quickly. The first aim is to get the lung back to normal as rapidly as possible, and early breathing exercises aid not only expansion, but direct drainage also. If the fluid is infected, whether purulent or not, it must be drained surgically at once, for delay involves damage to both pleura and lung. The suggested need for an Estlander operation should never arise if this principle be followed.

### Neurasthenia.

So much was this term abused that medical boards were instructed that attention need not be paid to it unless evident signs of nervous instability were present. The war has, however, demonstrated most emphatically that there is such a thing as nervous exhaustion, and the number of true neurasthenics has been very large. A thousand accessory causes contributed—bodily fatigue, training, shortage of food and drink, responsibility—neurasthenia was far more prevalent among the officers than among the rank and file—but the most potent has been mental fatigue. The author outlines the evidence of impending neurasthenia as irritability, difficulty in concentration, a tendency to start at a sudden sound, and a condition well described simply as jumpiness. Later sleep may come tardily, dreams become troublesome, and these lead to great nervous exhaustion, culminating in the complete breakdown which so many have seen in the active war zones. To a lesser degree this condition is seen in everyday life. Quiet, rest, and sleep, obtained, if possible, without drugs, are essentials. Inspired confidence and gradual return to the normal views of life are obtained by turning the mind from the past. Sir W. Hale-White rightly, we think, remarks that recent experience ought to have "made all doctors more interested in neurasthenia than some have been. It has been said that in ordinary civil practice the absence of organic symptoms, the constant complaining with apparently little justification, the frequent relapses alternating with recovery up to a certain point, the time and attention needed for their cure, has made some doctors neglect their neurasthenic patients, many of whom have been set down as malingerers or as sufferers from imaginary complaints."

